# Supplementary material for: Acceptance of COVID-19 Vaccination among Healthcare and Non-Healthcare Workers of Hospitals and Outpatient Clinics in the Northern Region of Slovakia
Source: Int J Environ Res Public Health. 2021 Dec 2;18(23):12695. doi: 10.3390/ijerph182312695 (PMC8657382; doi:10.3390/ijerph182312695)
Supplement: Supplementary file 1 [file ijerph-18-12695-s001.zip › ijerph-1456014-supplementary.pdf]

Supplementary Material, Table S1

ACCEPTANCE OF COVID-19 VACCINATION AMONG HEALTHCARE AND NON-HEALTHCARE WORKERS OF HOSPITALS AND OUTPATIENT CLINICS IN THE NORTHERN REGION OF SLOVAKIA

Table S1. The questionnaire.

Common part for vaccinated and unvaccinated:

Sex: male / female

Age (years): …………… Job duration (years): ……….

What is your job type?
Physician / Non-physician (but healthcare worker) / Non-healthcare worker

You are an employee of: hospital / outpatient clinic

Do you suffer from a chronic disease? Yes / No

If you suffer from a chronic disease, what is it?

- cardiovascular disease
- arterial hypertension
- kidney disease
- liver disease
- respiratory tract disease
- immune system disease
- diabetes mellitus
- endocrine system disease
- anaemia
- other

Have you undergone COVID-19? Yes / No

If you have undergone COVID-19, your work incapacity ended on (date): ………….

Have you ever been vaccinated against seasonal influenza? Yes / No

If you have been vaccinated against seasonal influenza, your last vaccination was (year): ………..

Try to assess your overall fear of COVID-19 (its course, consequences...) on a scale of 1-10:
1 – I am not afraid of COVID-19; 10 – I have serious concerns about COVID-19

Attitude/opinion on the introduction of compulsory vaccination for healthcare workers against COVID-19:

- Yes, I am in favor of introducing compulsory vaccination
- No, vaccination should be voluntary

Attitude/opinion on the introduction of compulsory vaccination for selected groups of population against COVID-19:

- Yes, I am in favor of introducing compulsory vaccination
- No, vaccination should be voluntary

Attitude/opinion on the introduction of compulsory vaccination for all population against COVID-19:

- Yes, I am in favor of introducing compulsory vaccination
- No, vaccination should be voluntary

Have you been vaccinated against COVID-19? Yes / No

Version for the vaccinated:
I was motivated to get vaccinated against COVID-19 by (choose any number of options):

- Concerns about COVID-19 itself
- An effort to prevent the spread of COVID-19 during the performance of my profession
- An effort to protect family members
- Being exempted from restrictive anti-epidemic measures after vaccination
- Other

What COVID-19 vaccine have you been vaccinated with:

- Comirnaty (Pfizer a BioNTech)
- Spikevax (Moderna)
- Vaxzevria (AstraZeneca)
- Janssen (Johnson&Johnson)
- Sputnik V (Gamaleya institute)

Version for the unvaccinated:
Reasons why I haven't been vaccinated against COVID-19 yet (choose any number of options):

- I am not afraid of COVID-19 – its course and consequences
- I do not find getting infected with COVID-19 likely
- I do not trust the efficacy of vaccines against COVID-19
- I have concerns about the safety and side effects of vaccines against COVID-19
- I went through COVID-19 (and assume lasting immunity against the disease)
- I have contraindications or expect a complicated vaccination course in my case
- Other
